# Exposure to second-hand tobacco smoke and respiratory symptoms in non-smoking adults: cross-sectional data from the general population of Telemark, Norway

**DOI:** 10.1186/s12889-018-5771-4

**Published:** 2018-07-06

**Authors:** A. K. M. Fell, M. V. Svendsen, J.-L. Kim, R. Abrahamsen, P. K. Henneberger, K. Torén, P. D. Blanc, J. Kongerud

**Affiliations:** 1 Department of Occupational and Environmental Medicine, Telemark Hospital, P. box 2900, Kjørbekk, 3710 Skien, Norway; 2 Department of Occupational and Environmental Medicine, Institute of Medicine, The Sahlgrenska Academy, University of Gothenburg, Gothenburg, Sweden; 3 Institute of Clinical Medicine, Faculty of Medicine, University of Oslo, Oslo, Norway; 4 National Institute for Occupational Safety and Health (NIOSH), Respiratory Health Division, Morgantown, WV USA; 5 Division of Occupational and Environmental Medicine, Department of Medicine, University of California, San Francisco, USCF, San Francisco, CA USA; 6 Department of Respiratory Medicine, Oslo University Hospital, Oslo, Norway

**Keywords:** Respiratory disease, Epidemiology questionnaire, Second-hand smoke, VGDF exposure

## Abstract

**Background:**

In Norway, data on the association between second-hand tobacco smoke (SHS) exposure at home and respiratory symptoms in adults are limited.

**Methods:**

We assessed the association between self-reported exposure to SHS and the prevalence of respiratory symptoms among never-smokers aged 16 to 50 years from the general population who were included in a cross-sectional population-based study in Telemark County, Norway. Logistic regression analysis was used to estimate the odds ratios of symptoms among 8850 never-smokers who provided an affirmative response to questions regarding SHS; 504 (5.7%) of these reported that they lived in a home with daily or occasional indoor smoking.

**Results:**

Productive cough and nocturnal dyspnoea were statistically associated with daily SHS exposure (ORs 1.5 [95% CI 1.04–2.0] and 1.8 [1.2–2.7], respectively). In analyses stratified by gender, nocturnal dyspnoea was associated with SHS among women (OR 1.8 [1.1–3.1]), but not among men (OR 0.93 [0.49–1.8]). Symptoms were not associated with occasional SHS exposure in the entire group, but infrequent exposure among men only was associated with increased prevalence of chronic cough; (OR 1.6; [1.04–2.6]) and was negatively associated with wheeze; (OR 0.44 [0.21–0.92)].

**Conclusions:**

Daily SHS exposure in private homes was associated with productive cough and nocturnal dyspnoea. Our results suggest that preventive measures may be needed to reduce the respiratory effects of SHS at home.

**Trial registration:**

ClinicalTrials.gov Identifier: NCT02073708 Registered February 27. 2014.

## Background

Exposure to second-hand tobacco smoke (SHS) is an established risk factor for non-malignant respiratory disease, especially among children, and also for cancer in adults [1–4]. Studies that have investigated respiratory effects among children exposed to SHS also have reported an increased risk of later developing chronic obstructive pulmonary disease in adulthood [2, 5]. However, knowledge about the association between SHS exposure in households and non-malignant respiratory effects in adults is limited [6–10], relatively few studies have restricted their analyses to never-smokers [7, 10, 11], or stratified analyses by gender [6, 11].

In March 2004, Ireland was the first European country to introduce smoking bans in all public restaurants and bars. Italy and Norway then followed suit. Since then, many countries have implemented smoking bans. A recent review of 77 studies assessing health effects of legislation controlling tobacco smoking in 21 countries concluded that, despite the consistent evidence of positive effects of national smoking bans on improving cardiovascular outcomes, the effects on respiratory health are less consistent [12]. The only Norwegian study included in that review was a study using a birth record registry. It reported fewer low birth weight and pre-term births in mothers who worked in bars and restaurants after enactment of the legislation.

Worldwide educational campaigns and interventions have been implemented to increase awareness of the adverse effects of SHS [12, 13]. Norway has used such nationwide campaigns since 1995. Lund and co-workers showed that after such a campaign attitudes to and awareness of the health risks of SHS were significantly increased in households containing smokers but that the prevalence of smoking was reduced only marginally [14]. In the late 1990s to early 2000s, exposure of children at home to SHS in Norway decreased from 32 to 18%. In 2004, a smoking regulation was enacted that prohibited smoking in all indoor public places in Norway including, but not limited to, workplaces [15]. The smoking ban in workplaces and public places has meant that for the most part indoor SHS exposure currently is limited to private household environments.

We aimed to investigate the association between SHS exposure in private homes and respiratory symptoms in a sample of never-smoking adults from the general population in Telemark County, Norway, 10 years after its introduction of a smoking ban in public places.

## Methods

### Study population

The current analyses use a subset of data from the Telemark study, which is a population-based study from Telemark County in south-east Norway. Specifically, the data are cross-sectional survey responses from never-smokers from the first questionnaire wave of that study carried out in 2013. The Telemark study, which has been described in detail elsewhere [1, 6], was based on a random sample of 50,000 subjects aged 16–50 years living in Telemark County, 48,142 of whom were traceable through a postal address. Of these, 16,099 completed a survey questionnaire (response rate 33%). A sample of non-responders found similar prevalence rates for respiratory symptoms and asthma compared to responders [16]. The majority of Telemark county’s approximately 170,000 inhabitants live in areas that are urbanized (but not heavily so), with the remainder being rural dwellers. This pattern is typical of Norway. The housing of the survey responders was distributed as follows: 72% detached houses, 10% semi-detached, and 18% apartments. Among the female responders, 23% were current smokers (including occasional smoking); 21% past smokers; and 56% never smokers. For males the corresponding proportions s were 24% current; 20% past smokers; and 56% never smokers. The characteristics of the subset of 8850 respondents who had never smoked and were included in the present analyses are shown in Table 1.

**Table 1:** Population characteristics of the 8850 Telemark study respondents who had never smoked

	All participants *N* = 8850	Female *N* = 4944	Male *N* = 3906
	*N* (%)	*N* (%)	*N* (%)
Age (years)			
16–30	3575 (40.4)	1992 (40.3)	1583 (40.5)
31–40	2132 (24.1)	1252 (25.3)	880 (22.5)
41–50	3143 (35.5)	1700 (34.4)	1443 (36.9)
Education			
Elementary school and additional 1–2 years (low)	1430 (16.2)	759 (15.4)	671 (17.2)
Upper secondary or certificate (medium)	3168 (35.1)	1536 (31.1)	1632 (41.8)
University (≥4 years) (high)	3905 (44.1)	2483 (50.2)	1422 (36.4)
Other or missing	347 (3.9)	166 (3.4)	181 (4.6)
Parental smoking during childhood	4557 (51.5)	2558 (51.7)	1999 (51.2)
Occupational exposure to VGDF^a^	3443 (38.9)	1313 (26.6)	2130 (54.5)
Exposure to mould or damp at home^b^	330 (3.7)	200 (4.0)	130 (3.3)

^a^Self-reported occupational exposure to vapour, gas, dust or fumes

^b^Self-reported exposure to mould or damp during the past 12 months

### Exposure

Second-hand tobacco smoking exposure at home was defined as an affirmative answer to the survey item: “Does anyone smoke tobacco in your current home?” followed by frequency options of: almost daily; 1–4 times/week; 1–3 times/month; or never. We collapsed responses into three categories: daily: occasional (1–4 times/week or 1–3 times/month), and never. Parental smoking with SHS exposure in childhood was defined by an affirmative answer to any one of three questions: “Was your mother a regular smoker when you were a child?”; “Was your father a regular smoker when you were a child?”; or “Were there any other regular smokers in your home when you were a child?” Occupational exposure to vapour, gas, dust or fumes (VGDF) was assessed by the question: “Have you been exposed to gas, smoke, dust or fumes in your work?” Exposure to mould and damp at home was defined by a positive response to one of the following: “Have you had any of the following in your residence: water damage/damage from damp inside the dwelling on walls, floors or ceilings, warped plastic mats, yellowed plastic coating or wood flooring that has become dark due to moisture; or visible mould on walls, floors or ceilings?” or “Have you at any time over the course of the past 10 years seen signs of moisture damage, water leakage or mildew in your home?”. Only those further affirming that exposure to damp or mould had occurred in the past 12 months were categorized as exposed.

Respondents with likely SHS exposure at work were identified by the application of an asthma-specific job-exposure matrix adapted for the Nordic countries (the N-JEM) [17]. The N-JEM defines an occupation as involving exposure to SHS based on the assumption that at least half of the subjects with this specific occupation would have a high probability of being exposed to SHS at work.

### Health outcomes

Respiratory health outcomes were defined based on the following survey items: asthma: “Do you have, or have you ever had asthma?”; physician-diagnosed asthma: “Has a doctor ever diagnosed you with asthma?”; ever wheezing: “Have you ever had whistling or wheezing in the chest?”; chronic cough: “Have you during the last years had a prolonged cough?”; productive cough: “Do you usually cough up phlegm or have mucus in the lungs that is hard to get up?”; nasal symptoms: “Have you ever experienced nasal symptoms such as stuffy nose, runny nose or sneeze attacks without having a cold?”; and current use of asthma medication: “Do you currently use any medication (spray, inhalation powder or tablets) for asthma?” In addition, symptoms in the past 12 months were defined by an affirmative answer to questions querying: wheezing or whistling in the chest; waking up with chest tightness cough, dyspnoea (three separate items), asthma attack; shortness of breath with wheezing, and wheezing without a cold. A symptom score was calculated by summing the number of affirmative answers to these questions and the question regarding the use of asthma medication (maximum symptom score = 8).

### Statistics

Statistical analyses were performed using IBM SPSS Statistics for Windows, version 23. Missing answers were coded as “no” for all variables. The Pearson chi-square test was used to test the difference in frequency for each symptom by SHS exposure status. Negative binomial regression analysis was used to assess the association between SHS and the symptom score. Dummy variables were used for occasional and daily SHS exposure. Logistic regression was used to estimate the odds ratio (OR) and 95% confidence interval (CI) of symptoms associated with SHS exposure adjusted for age, gender, educational level, exposure to mould and damp, parental smoking during childhood, and occupational VGDF exposure. Analyses were also repeated stratified by gender.

## Results

Among 8850 never-smokers, 504 (5.7%) stated that they currently lived in a household with daily or occasional indoor smoking. Previous parental smoking was reported by 4557 (51.5%), current or past exposure to occupational exposure to VGDF by 3443 (38.9%), and visible mould or damp at home in the previous year by 330 (3.7%). The frequency of respiratory symptoms by SHS exposure status is shown in Table 2. Dyspnoea with wheeze, wheezing without a cold, and nocturnal chest tightness were reported by 10–12% of respondents; night-time cough was approximately twice as common, being reported by 21% of respondents.

**Table 2:** Respiratory symptom frequency grouped by SHS exposure at home for all participants and by gender

Symptoms or disease	Exposure to second-hand tobacco smoke at home*								
	All participants (*N* = 8850)			Women (*N* = 4944)			Men (*N* = 3906)		
	Never (*N* = 8346)	Occasionally (*N* = 248)	Daily (*N* = 256)	Never (*N* = 4656)	Occasionally (*N* = 140)	Daily (*N* = 148)	Never (*N* = 3690)	Occasionally (*N* = 108)	Daily (*N* = 108)
	*n* (%)	*n* (%)	*n* (%)	*n* (%)	*n* (%)	*n* (%)	*n* (%)	*n* (%)	*n* (%)
Ever had symptom									
Asthma	1044 (13)	36 (15)	37 (15)	597 (13)	22 (16)	21 (14)	447 (12)	14 (13)	16 (15)
Physician-diagnosed asthma	910 (11)	34 (14)	32 (13)	515 (11)	20 (14)	18 (12)	395 (11)	14 (13)	14 (13)
Wheezing	1774 (21)	55 (22)	53 (21)	1023 (22)	29 (21)	38 (26)	751 (20)	26 (24)	15 (14)
Chronic cough	1546 (19)	55 (22)	50 (20)	928 (20)	28 (20)	31 (21)	618 (17)	27 (25)	19 (18)
Productive cough	**924 (11)**	**29 (12)**	**46 (18)**	**535 (12)**	**19 (14)**	**28 (19)**	389 (11)	10 (9)	18 (17)
Nasal symptoms without cold	3580 (43)	96 (39)	112 (44)	2055 (44)	53 (38)	69 (47)	1525 (41)	43 (40)	43 (40)
Past 12 months									
Use of asthma medication	569 (7)	19 (8)	17 (7)	333 (7)	14 (10)	14 (10)	236 (6)	5 (5)	3 (3)
Wheezing	1288 (15)	34 (14)	45 (18)	748 (16)	26 (19)	32 (22)	540 (15)	8 (7)	13 (12)
Nocturnal chest tightness	**970 (12)**	**39 (16)**	**30 (15)**	**615 (13)**	**26 (19)**	**28 (19)**	355 (10)	13 (12)	11 (10)
Nocturnal cough	1781 (21)	55 (22)	62 (24)	1220 (26)	35 (25)	44 (30)	561 (15)	20 (19)	18 (17)
Asthma attack	312 (4)	14 (6)	14 (6)	201 (4)	**12 (9)**	**10 (7)**	111 (3)	2 (2)	4 (4)
Dyspnoea while wheezing	939 (11)	29 (12)	32 (13)	547 (12)	19 (14)	24 (16)	392 (11)	10 (9)	8 (7)
Wheezing without cold	844 (10)	28 (11)	30 (12)	476 (10)	18 (13)	23 (16)	368 (10)	10 (9)	7 (7)
Nocturnal dyspnoea	**477 (6)**	**18 (7)**	**28 (11)**	**286 (6)**	**13 (9)**	**18 (12)**	191 (5)	5 (5)	10 (9)
Mean symptom score (SD)^a^	0.86 (1.6)	0.95 (1.8)	1.04 (1.8)	**0.95 (1.7)**	**1.2 (2.0)**	**1.3 (1.9)**	0.75 (1.5)	0.68 (1.4)	0.69 (1.5)

*p-value based on comparison to Never category < 0.05 in bold; second-hand smoking at home: Daily: almost daily; Occasional: (1–4 times/week or 1–3 times/month

^a^SD: standard deviation

No respiratory symptoms during the past 12 months were reported by 5892 respondents (66.6%). One, two, three, four, five, six, seven or eight symptoms were reported by 1239 (14.0%), 508 (5.7%), 419 (4.7%), 292 (3.3%), 220 (2.5%), 130 (1.5%), 77 (0.9%) and 73 (0.8%), respectively.

We examined the association between SHS and individual symptoms adjusting for gender (except in the gender stratified analyses), age, educational level, exposure to vapour, gas, dust or fumes at any job, mould or damp exposure at home in the past 12 months, and parental SHS during childhood (Table 3). The prevalence of productive cough (ever) and nocturnal dyspnoea (last 12 months) were the only two symptoms statistically associated with daily SHS exposure (ORs 1.5 [95% CI 1.04–2.0] and 1.8 [1.2–2.7], respectively). In analyses stratified by gender, the association of daily SHS with productive cough was similar for women and men (ORs 1.4 and 1.5, respectively, neither point estimate excluding 1.0) (Table 3). In contrast, stratified by gender, nocturnal dyspnoea was associated with SHS among women; OR 1.8 (CI 1.1–3.1), but not among men; OR 0.93 (0.49–1.8). No symptom was statistically associated with occasional SHS exposure in the entire group, although occasional exposure among men only was associated with significantly increased prevalence of ever having chronic cough; OR 1.6 (1.0–2.6) and was negatively associated with ever having wheeze; OR 0.44 (0.21–0.92). In negative binomial regression models that adjusted for potential confounders, the symptom score was not statistically associated with neither occasional nor daily SHS.

**Table 3:** Respiratory symptoms and exposure to SHS at home grouped by gender, adjusted^a^ odds ratios

Symptoms or disease	Exposure to second-hand tobacco smoke at home^†^						
	All participants (*N* = 8850)			Women (*N* = 4944)		Men (*N* = 3906)	
	Never (*N* = 8346)	Occasional (*N* = 248)	Daily (*N* = 256)	Occasional (*N* = 140)	Daily (*N* = 148)	Occasional (*N* = 108)	Daily (*N* = 108)
Ever had symptom							
Asthma	1.0	1.2 (0.80–1.7)	1.1 (0.77–1.6)	1.2 (0.73–1.9)	1.1 (0.66–1.7)	1.1 (0.60–1.9)	1.1 (0.65–2.0)
Physician-diagnosed asthma	1.0	1.3 (0.87–1.8)	1.1 (0.74–1.6)	1.3 (0.80–2.1)	1.1 (0.64–1.8)	1.2 (0.68–2.1)	1.1 (0.61–2.0)
Wheezing	1.0	1.0 (0.76–1.4)	0.93 (0.68–1.3)	0.90 (0.59–1.4)	1.2 (0.82–1.8)	1.2 (0.78–1.9)	0.61 (0.35–1.1)
Chronic cough	1.0	1.2 (0.91–1.7)	1.1 (0.77–1.5)	1.0 (0.65–1.5)	1.1 (0.71–1.6)	**1.6 (1.04–2.6)**	1.1 (0.64–1.8)
Productive cough	1.0	0.93 (0.63–1.4)	**1.5 (1.04–2.0)**	1.0 (0.6–1.7)	1.4 (0.93–2.2)	0.80 (0.41–1.5)	1.5 (0.89–2.6)
Nasal symptoms without cold	1.0	0.84 (0.65–1.1)	1.1 (0.82–1.4)	0.79 (0.56–1.1)	1.2 (0.82–1.6)	0.93 (0.62–1.4)	0.94 (0.63–1.4)
Past 12 months							
Use of asthma medication	1.0	1.0 (0.64–1.7)	0.8 (0.49–1.4)	1.2 (0.70–2.2)	1.1 (0.61–1.9)	0.67 (0.27–1.7)	0.38 (0.12–1.2)
Wheezing	1.0	0.81 (0.56–1.2)	1.0 (0.75–1.5)	1.1 (0.69–1.7)	1.3 (0.83–1.9)	**0.44 (0.21–0.92)**	0.75 (0.42–1.4)
Nocturnal chest tightness	1.0	1.3 (0.89–1.8)	1.2 (0.8–1.7)	1.3 (0.85–2.1)	1.3 (0.85–2.1)	1.2 (0.64–2.1)	0.93 (0.49–1.8)
Nocturnal cough	1.0	0.98 (0.72–1.3)	1.1 (0.79–1.4)	0.86 (0.58–1.3)	1.1 (0.74–1.5)	1.2 (0.73–2.0)	1.1 (0.63–1.8)
Asthma attack	1.0	1.4 (0.83–2.5)	1.3 (0.76–2.3)	1.8 (0.98–3.4)	1.3 (0.67–2.6)	0.61 (0.15–2.5)	1.3 (0.46–3.7)
Dyspnoea while wheezing	1.0	0.96 (0.64–1.4)	0.99 (0.68–1.5)	1.1 (0.65–1.8)	1.3 (0.71–1.6)	0.77 (0.40–1.5)	0.59 (0.28–1.2)
Wheezing without cold	1.0	1.0 (0.70–1.6)	1.0 (0.71–1.6)	1.2 (0.69–1.9)	1.4 (0.86–2.2)	0.87 (0.44–1.7)	0.38 (0.12–1.2)
Nocturnal dyspnoea	1.0	1.2 (0.71–1.9)	**1.8 (1.2–2.7)**	1.4 (0.77–2.5)	**1.8 (1.1–3.1)**	0.79 (0.32–2.0)	0.93 (0.49–1.8)
Symptom score^‡^		0.056 (−0.13–0.24)	0.13 (−0.050–0.31)	0.097 (−0.14–0.33)	0.21 (−0.010–0.44)	−0.12 (−0.42–0.19)	−0.11 (−0.42–0.19)

^a^Adjusted for age, gender (in the unstratified analyses), education, ever exposure to vapour, gas, dust or fumes at work, mould or damp exposure at home in the past 12 months and parental SHS during childhood

^†^*p*-value based on comparison to Never category < 0.05 in bold; second-hand smoking at home: Daily: almost daily; Occasional: (1–4 times/week or 1–3 times/month

^b^Negative binominal regression with regression coefficients and 95% confidence interval

Further, exclusion of those stating SHS exposure one to three times per month or combining the SHS exposures occasionally and daily into one category did not change these results substantially (data not shown). Regression analyses were also performed including 129 participants (1.4%) identified through the N-JEM as being exposed to SHS at work and the results were similar (data not shown).

## Discussion

In this study, the prevalence of productive cough and nocturnal dyspnoea were associated with frequent SHS exposure at home among never-smokers. These associations took into account demographic, environmental and occupational covariates. In contrast, SHS exposure was not associated with a composite symptom score.

The prevalence of respiratory symptoms in the past 12 months we observed is similar to those reported among non-smokers in a similar study from Sweden [18], which found that 8–23% experienced wheezing, nocturnal, or chronic cough or nasal symptoms. This is also consistent with the findings of other studies of the general population in Norway and confirms that respiratory symptoms are prevalent in adults [19].

Relatively few studies have assessed SHS exposure in private homes among never-smokers. Two early studies of this question were published in 1994 [10, 20]. Leuenberger et al. included 4194 people aged 18–60 years in Switzerland and found associations between SHS exposure and elevated odds of bronchitis symptoms (OR 1.59 [95% CI 1.17–2.15]), symptoms of chronic bronchitis (OR 1.65 [1.28–2.16)) and dyspnoea (OR 1.45 [1.20–1.76]). Unlike in our study, wheezing without a cold (OR 1.94 [1.39–2.70]) and physician-diagnosed asthma (OR 1.39 [1.04–1.86]) also were associated with SHS exposure. While childhood SHS most likely does effect respiratory health, it may be difficult for participants to accurately estimate both the regularity and degree of such exposure in retrospect. This may result in overestimation of the prevalence and, in turn, diminish the estimated effect of this exposure.

In the second study, Dayal et al. included 4200 never-smokers in Philadelphia. They found that the prevalence of obstructive respiratory disease (defined as asthma, chronic bronchitis or emphysema) was proportional to the level of SHS (OR 1.86 [1.21–2.86]) in the home and roughly equivalent to directly smoking > 1 pack of cigarettes per day. Two later studies from California, but also from the 1990s, assessed non-current smokers (never smokers and ex-smokers), reporting a relationship between self-reported SHS exposure and increased risk of asthma, chronic bronchitis or emphysema, chronic bronchitis symptoms, and airway obstruction identified by pulmonary function testing [8, 21]. Interpretation of these previous studies must be tempered, however, by the recognition that smokers smoked more cigarettes per day in the 1990s and thus might have carried greater health impacts for those with SHS exposure [22, 23].

More recently, the European Community Respiratory Health Survey (ECRHS) also assessed the effects of SHS exposure among never-smoking adults (*n* = 7882) [7]. In that study, SHS exposure at home and at work combined was associated with nocturnal chest tightness (OR 1.3 [95% CI 1.0–1.6]), nocturnal breathlessness (OR 1.3 [CI 1.1–1.7]) and breathlessness after activity (OR 1.3 [1.1–1.5]). As in our study, there was no association between household SHS and adult asthma. The full extent to which household SHS may or may not be associated asthma or other airway conditions in adults will require prospective studies for elucidation.

Importantly and in contrast to our study, in analyses restricted to home SHS exposure only, the ECRHS study did not identify an association with respiratory symptoms. To test whether our results were altered by the inclusion of subjects with occupational SHS exposure (as in the ECHRS example), we included the 129 respondents who were exposed to SHS at work based on the N-JEM. The findings including these subjects were similar to the main analyses. Because few respondents were assessed as having SHS exposure at work in the Telemark study, the power was not sufficient to detect effects for this group separately.

When we stratified by gender, we observed an SHS association with nocturnal dyspnoea in the past 12 months only among women. The male-specific findings were limited to occasional but not daily SHS, thus inconsistent with an exposure gradient effect. Indeed, we were unable to clearly demonstrate a pattern of step-up in SHS effect in men and women combined, for example for productive cough (occasional SHS OR = 0.93, daily SHS OR = 1.5). Nevertheless, there was a suggestion of a step-up for nocturnal dyspnoea (occasional OR = 1.2, daily OR = 1.8) that was even more evident among the female stratum (occasional 1.4; daily 1.8). Albeit limited, some of the epidemiological literature also supports gender differences in the association between SHS exposure and respiratory health, with an effect more apparent among women. A study from China assessed lung function among 1033 adults aged 40–69 years and included both ever- and never-smokers, and found a reduction in lung function associated with SHS among women only [6]. A recent study among 3568 old-order Amish in the U.S. found that exposure to second-hand tobacco smoke was associated with a 2.7% lower forced expiratory volume in 1 sec (FEV_1_) percentage in women [24].

We defined SHS based on conditions in a participant’s current home. This could differ systematically from a prior level of SHS exposure experienced in relation to the presence or absence of an adverse health outcome. This is particularly relevant to symptoms a participant ever had versus in the past 12 months. For example, the male-specific finding of ever chronic cough associated with occasional SHS may reflect current reduction in exposure prompted by prior symptoms. This temporal limitation should also be taken into account in interpreting the association of daily SHS with ever having a productive cough, but should not apply to results for nocturnal dyspnoea in the past 12 months.

A few other studies have assessed the effects of SHS exposure on lung function. A Norwegian study by Skogstad and co-workers showed that employees of restaurants and bars (*n* = 69) had significant cross-shift changes in lung function indices associated with SHS exposure [25]. The cross-shift reduction in FVC changed from 81 ml (standard deviation (SD) = 136) during exposure to SHS to 52 ml (SD = 156) (*p* = 0.24) following the smoking ban. The reduction in FEV_1_ during a work-shift was borderline significantly reduced from 89 ml (SD = 132) to 46 ml (SD = 152) (*p* = 0.09). A larger decrease in FEV_1_ was observed in non-smokers and participants with asthma from the pre-ban work-shift to the post-ban work-shift. A French survey compared the spirometric measurements of two groups of non-smokers: those with and without exposure to passive smoking at home [9]. The authors found that non-smoking subjects of either sex whose spouse was a current smoker of at least 10 g of tobacco a day had significantly lower forced mid-expiratory flow rate (FEF_25–75%_) than did those married to non-smokers. Women also showed a significant difference in FEV_1_ and a clear dose–response effect related to the amount of smoking by their husbands.

Our study has important limitations that should be kept in view. We did not distinguish between smoking at home that was unlimited or, in contrast, limited (for example, smoking only in one room, next to an open window, or restricted to a balcony). This potential exposure misclassification is mitigated by standard Norwegian usage in which smoking on the balcony or by an open window typically would not be understood as constituting smoking “inside the home.” Another potential study limitation is that we did not ascertain other sources of indoor air pollution such as from wood stoves, fireplaces or unventilated home cooking. Such sources would have to have been systematically linked to SHS exposure, however, to have confounded the associations that we observed.

The lack of a firm temporal sequence for some of the symptoms has already been alluded to. The low study response rate also must be considered. However, we addressed non-participation and performed inverse probability weighting to account for non-response bias in a separate study [16]. In that study, we reported demographic characteristics, respiratory symptoms, and use of asthma medication for the non-responders and assessed possible selection bias. A total of 260 of 700 randomly selected non-responders (37%) participated in the non-responder study. No significant differences were detected for the prevalence of asthma and several respiratory symptoms between responders and non-responders in the Telemark study. The prevalence of cough and use of asthma medication was slightly higher in responders, and thus we performed later analyses with and without weighted data sets, but found that the weighting had little effect on the study outcomes [26]. Comparison of the results between responders and non-responders suggests that current smoking as a risk factor for productive and chronic cough may have been underestimated in the Telemark study. This is not relevant in the current study, however, because it is limited to never-smokers. Unfortunately, information on SHS exposure in non-responders was not available. The non-response analyses also detected that occupational exposure to VGDF as a risk factor for respiratory symptoms may be overestimated in the Telemark study. This difference in exposure–outcome associations emphasizes the importance of assessing occupational exposure to VGDF as a potential confounder, as we have done in the current analyses. Although the non-response assessments in the Telemark study demonstrated that exposure-outcome associations were not affected by non-response, results may not be entirely representative of the initial population. In addition to these limitations, it also should be noted that the small number of respondents with some of the health outcomes (e.g., asthma attacks and nocturnal dyspnoea) accounts for the relatively wide confidence intervals for certain OR estimates.

In this study, we used self-reported second-hand smoking in private homes as the exposure metric and had no biological monitoring of exposure, which could have resulted in exposure misclassification. Correlation between self-report of SHS exposure and biomarker levels (e.g., cotinine) has been reported [27–31], although there also are other studies showing lack of correlation or discrepancies between self-report and objective biomarkers of exposure [32, 33]. Hence, selective misclassification cannot be excluded. The possibility that never-smokers with respiratory symptoms are more likely to remember SHS exposure than are those without symptoms may lead to an overestimation of effects. Direct biomonitoring of SHS levels is difficult because cotinine reflects exposure only during the past few days [30]. Other personal measurements of nicotine (including in hair) provide an important alternative metric that, unfortunately, was not available in our study protocol [31, 34].

Despite these limitations, our findings suggest that SHS in private homes may affect the respiratory health of never-smokers at least in terms of selected symptoms even though, as in any cross-sectional study, no causal inferences can be conclusively drawn. Longitudinal studies that include biomonitoring will be needed to show such effects convincingly. Nevertheless, our findings do support the potential benefits of extending smoke-free environments from public spaces to private homes, as others have suggested as well [35].

## Conclusion

While adult consequences of long-term SHS exposure are known, this cross-sectional population-based study from Telemark, Norway, provides additional information about the short- or intermediate-term consequences that non-smokers may experience.

## Abbreviations

CI: 95% confidence interval
ECRHS: European community respiratory health survey
FEF_25–75%_: Forced mid-expiratory flow rate
FEV_1_: Forced expiratory volume in one second
FVC: Forced vital capacity
N-JEM: Asthma-specific job-exposure matrix adapted for the Nordic countries
OR: Odds ratio
SD: Standard deviation
SE: Standard error
SHS: Second-hand tobacco smoking
VGDF: Vapour, gas, dust and fumes
